# Black Americans demonstrate comparatively low levels of depression and anxiety during the COVID-19 pandemic

**DOI:** 10.1371/journal.pone.0253654

**Published:** 2021-06-25

**Authors:** Victoria Owens, Htay-Wah Saw

**Affiliations:** 1 Joint Program in Survey Methodology (JPSM), University of Maryland & Westat, College Park, Maryland, United States of America; 2 Michigan Program in Survey Methodology (MPSM), Survey Research Center (SRC), Institute for Social Research (ISR), University of Michigan, Ann Arbor, Michigan, United States of America; 3 Center for Economic and Social Research (CESR), University of Southern California (USC), Los Angeles, California, United States of America; CentERdata, NETHERLANDS

## Abstract

**Introduction:**

During public health crises like the COVID-19 pandemic, populations can experience worsening mental health. Prior reports have suggested that Black Americans experienced lower rates of anxiety and depression than White Americans before the pandemic; however, during the pandemic, outcomes may be different as Black Americans have been disproportionately affected in terms of mortality, hospitalization, COVID-19 infection, and job loss. We documented the differential mental health impact of COVID-19 on Black and Non-Black Americans.

**Methods:**

We analyzed nationally representative longitudinal data from the Understanding America Study COVID-19 Tracking Survey spanning March through November of 2020 to assess differences over time in prevalence of anxiety and depression between Black and non-Black Americans.

**Results:**

We found that Black Americans were significantly less likely to report symptoms for anxiety, depression, or both during the pandemic. In a given month between March through November of 2020, the odds of Black Americans reporting such symptoms was on average about half that of Non-Black Americans. We also found that in September 2020, the gap in reporting symptoms for depression began to widen gradually. Specifically, since that time, prevalence of depression remained stable among non-Black Americans while it declined gradually among Black Americans. Our main results were robust to adjusting for demographics, risk perceptions, and baseline pre-pandemic mental health status.

**Conclusions:**

Black Americans maintained significantly better mental health than Non-Black Americans despite their struggle against economic, health, and racial inequalities during the pandemic. We discuss the significance and implications of our results and identify opportunities for future research.

## Introduction

Black Americans have faced disproportionately high infection rates, job losses, and loss of life during the COVID-19 pandemic. This population may be at greater risk of severe illness from COVID-19 due to higher prevalence of certain comorbidities compared to non-Black Americans, including high blood pressure and diabetes [1–3]. Indeed, 33% of non-Hispanic (NH) Black Americans, compared to 27% of NH White Americans, demonstrated risk factors for severe COVID-19 illness [4]. This has likely led to disproportionate rates of hospitalizations and deaths of NH Black Americans throughout the pandemic [5–7].

Economic inequalities along racial and ethnic lines, worsened by the pandemic, compound these health risks. The Bureau of Labor Statistics reported that higher unemployment rates among non-Hispanic Black Americans persisted through October of 2020 [8]. Black Americans also more often hold jobs demanding on-site work: only 19.7% of non-Hispanic Black Americans reported they had the ability to work from home in 2017–2018, compared to 16.2% of Hispanic Americans and 29.9% of non-Hispanic White Americans and 37.0% of non-Hispanic Asian Americans [9].

Intuitively, mental health impacts could follow the unequal disruptions of Black Americans’ physical and economic security. Two separate surveys utilizing non-probability panels with data weighted to U.S. demographic totals found higher rates of suicide consideration, substance use to cope with the pandemic [10], and suicide behavior risks [11] among Black than White Americans early in the pandemic. Another non-probability study conducted after the stock market drop in March 2020 reported higher levels of anxiety related to economic hardship were among Black Americans than their counterparts [12]. Despite this, no increase in symptoms of depression were observed in nationally representative probability surveys among Black Americans as lockdowns commenced in March and April 2020 [13].

Before the pandemic, Black Americans appeared to generally experience lower rates of anxiety and depression compared to other racial and ethnic groups, yet suffered higher prevalence of serious mental health issues. While they had lower lifetime rates of mood and anxiety disorders than White Americans [14] and lower 12-month prevalence of generalized anxiety disorder, panic disorder, and social anxiety, Black Americans were in contrast more likely to meet criteria for PTSD and to have serious psychological distress than White Americans [15, 16]. Even among poor Black Americans, life satisfaction is higher and stress incidence is lower compared to White Americans [17]. It is possible that this difference would continue after the onset of the pandemic, but on the other hand, mental health trends may not maintain the same pattern because of the aforementioned inequalities between racial and ethnic groups.

Mental health outcomes during the COVID-19 pandemic should inform public health policy. To our knowledge, the trajectory of both depression and anxiety for racial and ethnic groups during the pandemic in the U.S. have not yet been examined using longitudinal data. The ability to recover from anxiety and depression over time may differ among racial and ethnic groups, considering the differences in economic resources accessible to them during the pandemic. Thus, our primary aims are to (1) analyze differences in odds of having depression and anxiety among Black and non-Black U.S. adults when state lockdowns were first implemented in March; and (2) explore changes in odds of developing depression and anxiety among Black and non-Black U.S. adults over time between March and November 2020.

## Methods

### Procedures and methods

#### Data

Data for this study was from the Understanding America Study (UAS) [18]. Established at the University of Southern California (USC) in 2014, the UAS is a nationally representative, probability-based internet panel of U.S. households and consists of approximately 9,000 respondents aged 18 and above. Prior access to the internet is not a prerequisite to be in the panel; respondents without prior internet access are provided with a computer tablet and broadband internet subscription [19]. Respondents complete surveys once or twice a month covering various substantive topics. Partly as a result of this, the UAS comprises a vast amount of background information on UAS respondents, including extensive measures of physical and mental health, income, labor force participation, cognitive functioning, and demographics.

#### COVID-19 tracking survey

Since March 10, 2020, all active members of the UAS panel have been invited to participate in an ongoing coronavirus tracking survey [20]. Each UAS panel member participating in the tracking survey receives a survey every two weeks. Every day, about 500 respondents are invited to take the tracking survey for a total of about 7,000 respondents over a two-week period. The micro data, questionnaires, codebooks and reports are publicly available to any registered data users at the UAS data website [18]. As of November 30, 2020, a total of 17 survey waves have been completed. Mean response rate across all survey waves is 83.63% (SD = 6%; Min = 76.35%; Max = 96.66%).

### Measures

#### Mental health outcome measures

The mental health outcome was measured by the well-validated Patient Health Questionnaire-4 (PHQ-4), a brief 4-question survey developed by Kroenke et al. [21] and validated by Löwe et al. [22] (Cronbach’s *α* = 0.82) to screen patients for depression (frequency of “feeling, down, depressed or hopeless,” and “little interest or pleasure in doing things”) and anxiety (frequency of “feeling nervous, anxious, or on edge” and “not being able to stop or control worrying”) over the past fourteen days. Response options for each item included “not at all,” “several days,” “more than half the days,” and “nearly every day.” Scores across the four items can be summed up to analyze the overall mental health outcome. Alternatively, each condition (depression, anxiety) can be scored and analyzed separately. We considered three binary outcomes in this study: showing symptoms for both depression and anxiety disorder if scores on the overall scale are ≥ 6 (probable depression and anxiety); showing symptoms for depression disorder if the scores on depression subscale are ≥ 3 (probable depression); and showing symptoms for anxiety disorder if the scores on the anxiety subscale are ≥ 3 (probable anxiety; 22). These cutoff values are indicative of major depressive disorder and generalized anxiety disorder with high sensitivity and specificity [23, 24]. We include the combined measure of symptoms for anxiety and depression as an indicator of overall mental distress as prior research has done [25].

#### Race variable

Respondents reported their race according to one of the following response options: White only, Black only, American Indian or Alaska Native only, Asian only, Hawaiian/Pacific Islander only, or Mixed. Respondents also reported whether they are Spanish, Hispanic or Latino. For comparing the mental health outcome across racial groups, we used these two pieces of information to create five racial groups: NH White (White); NH Black (Black); NH Asian (Asian); NH Other (Other); and Hispanic. We combined Whites, Asians, Others, and Hispanics into one group for comparing Black Americans vs. non-Black Americans.

#### Baseline depression symptoms

In earlier waves of UAS surveys, respondents also completed the 8-item Center for Epidemiologic Studies–Depression Scale (CES-D), which entered our regressions as control for baseline depression symptoms [26]. Previous research has shown that CES-D scores are reasonably well-correlated with the PHQ-9, from which the PHQ-4 is derived [27]. Scores range from 0–8. We used the most recent CES-D data before the start of Wave 1 of UAS COVID-19 Tracking Survey in March 10, 2020, as some respondents completed the 8 CES-D items more than once as part of the ongoing Health and Retirement Study (HRS) that is fielded in the UAS every two years. The time gap between the latest pre-pandemic CES-D assessment and the start of Wave 1 of UAS COVID-19 Tracking Survey on March 10, 2020 is about 12.75 months (S.D = 8.07 months; min = 0.033 month; max = 58.53 months). This indicates that on average the CESD assessment was taken one year prior to the onset of COVID-19 in March, 2020. We also consider a mid-pandemic CES-D assessment taken in late December 2020.

#### Demographic variables

We also controlled for a set of covariates, which included: age (18–34, 35–54, 55–64, or 65+); highest educational attainment (high school diploma or below, some college, or college and above), annual household income ($0–29,999, $30–59,999, $60–99,999, or $100,000 or more), gender (female or male), whether a household has children under 18 or not, employment status (currently employed; currently unemployed; or others that included retired, individuals on sick leave, disabled, others). UAS respondents’ key demographic information are updated every 3 months. More details about how the UAS respondents’ demographic information are updated are available at the following website: (https://uasdata.usc.edu/index.php?r=eNpLtDK0qi62MrFSKkhMT1WyLrYytFwwskuTcjKT9XIrM_JLi1Mz8nNSlKxrASlLDmM). We use the most updated demographic information in the analysis. Information about employment status was collected in every wave of the UAS COVID-19 Tracking Survey since employment status changed more frequently for some subpopulation groups during the pandemic.

*Risk perceptions*. To check the robustness of our main results, we also considered whether adjusting for risk perceptions in the models would change the outcome. Particularly, we analyzed responses to the following three risk perception questions asked in the UAS COVID-19 Tracking survey: “What is the chance that you will get the coronavirus in the next three months?”; “If you do get infected with the coronavirus, what is the chance you will die from it?”; and “What is the percent chance that you will run out of money because of the coronavirus in the next three months?” Responses to the three questions were provided on a validated visual linear scale ranging from 0% to 100% [28].

*Data analyses*. All analyses applied survey weights, which adjusted for probabilities of sample selection and survey nonresponse; and aligned the UAS sample with distributions of key demographic variables in the U.S. civilian population in terms of age, gender, race/ethnicity, education, and location (see https://papers.ssrn.com/sol3/papers.cfm?abstract_id=3502405 for detailed information about how the UAS survey weights were constructed) [29]. All regression analyses control for the aforementioned demographic variables and baseline CES-D mental health score. We reported odds ratios, 95% CIs, and level of statistical significance for each logistic regression. Robust standard errors were used in cross-sectional logistic regressions, whereas cluster robust standard errors were used in longitudinal logistic regressions to allow for possible correlation in responses across survey waves within respondents. All analyses were conducted in 2020 using Stata version 15.0.

## Results

Table 1 presents weighted distributions of demographic characteristics of the UAS COVID sample (counts within subgroups are in parentheses). In Wave 1, 8,815 UAS respondents were invited. Of those, 7,145 completed the survey, 54 started but did not complete the survey, and 1,616 did not start the survey, yielding a response rate of 81.06%. We conducted non-response analysis and found a slight imbalance in some demographic characteristics between responders and non-responders; this difference was not big enough to alter the conclusion of our study findings. Wave 1 respondent and non-respondent data can be assessed at the following UAS data webpage: https://uasdata.usc.edu/index.php.

**Table 1:** UAS COVID sample demographic composition (N = 6,932) vs ACS estimates.

	UAS Sample (Weighted)	ACS Estimates (Weighted)
**Age**		
18–34 (1,435)	24%	30%
35–54 (2,586)	37%	32%
55–64 (1,406)	18%	17%
65+ (1,498)	20%	21%
**Education**		
High school diploma or GED and below (1,537)	38%	39%
Some college but no college degree (2,572)	28%	30%
College and above (2,823)	34%	31%
**Race**		
White (4,528)	63%	63%
Black (540)	12%	12%
Asian (343)	5%	6%
Other (368)	3%	3%
Hispanic (1,142)	17%	16%
**Income**		
$0–29,999 a year (1,680)	27%	19%
$30–59,999 a year (1,771)	27%	21%
$60–99,999 a year (1,686)	23%	24%
$100,000 or more a year (1,778)	23%	37%
**Gender**		
Female (4,063)	52%	51%
Male (2,869)	48%	49%
**Children**		
No children (4,718)	65%	66%
Have children (2,214)	35%	34%
**Employment**		
Employed (4,144)	59%	NA
Unemployed (543)	9%	NA
Others (2,245)	32%	NA
**Baseline depression symptoms**		
Baseline CES-D Score	Mean = 2.24 (SD = 2.01)	NA

For comparison, we also provide weighted distributions of the same characteristics from the 2019 American Community Survey (ACS) for U.S. adults aged 18 and above [30]. ACS data are 1-year estimates (https://data.census.gov/mdat/#/search?ds=ACSPUMS1Y2019). The ACS is conducted by the U.S. Census Bureau every month, every year, and samples about 3.5 million U.S. households a year to create accurate population estimates. Overall, the ages of sample respondents ranged from 18 to 101 years old with a mean age of 48 (in terms of age groups, 24% of sample respondents were 18–34, 37% were 35–54, 18% were 55–64, and 20% were 65+). About 38% had a high school diploma or GED or below, 28% went to college but did not earn a degree, and 34% earned at least a college degree. As for racial and ethnic composition, 63% identified themselves as White, 12% as Black, 5% as Asian, 3% as Other, and 17% as Hispanic. About 27% came from households with annual household income less than $30K, 27% between $30K-$60K, 23% between $60K-$100K, and 23% above $100K. 52% of sample respondents are female, with 65% coming from households with no children under 18. The mean baseline CES-D score was 2.24 (SD = 2.01; Min = 0; Max = 8) and did not differ significantly between Black Americans (2.27) and non-Black Americans (2.24) (p-value = 0.757). In contrast, the mean mid-pandemic CES-D score was 3.05 (SD = 1.66; Min = 0; Max = 8) and was significantly lower for Black Americans (2.91) than non-Black Americans (3.06; p-value = 0.04), showing that both Black and non-Black Americans began in a similar position in terms of depression, but then diverged after the onset of the pandemic. Table 2 presents distributions of the same demographic characteristic, split by non-Black vs. Black Americans.

**Table 2:** Sample demographic composition, split by non-Black and Black vs. Americans.

	Non-Black Americans (N = 6,381)	Black Americans (N = 540)	Significance (Test of difference between non-Black and Black Americans)
**Age**			
18–34	21%	20%	
35–54	37%	44%	***
55–64	20%	22%	
65+	22%	14%	***
**Education**			
High school diploma or GED and below	22%	30%	***
Some college but no college degree	37%	43%	***
College and above	42%	27%	***
**Income**			
$0–29,999 a year	22%	46%	***
$30–59,999 a year	25%	28%	
$60–99,999 a year	25%	14%	***
$100,000 or more a year	27%	12%	***
**Gender**			
Female	58%	70%	***
Male	42%	30%	***
**Children**			
No children	68%	68%	
Have children	32%	32%	
**Employment**			
Employed	60%	57%	
Unemployed	8%	12%	***
Others	32%	31%	
**Baseline depression symptoms**			
Baseline CES-D Score	2.24	2.27	

* p<0.10;

** p<0.05;

*** p<0.01

### Cross-sectional analysis

In Table 3, we presented the prevalence of probable anxiety and depression, anxiety, and depression for each of the subgroups. In Table 4, we present our estimates of adjusted odds ratios (AOR) for having probable anxiety and depression, anxiety, and depression. Data are from Wave 1 of the UAS COVID Tracking Survey, fielded between March 10–30, 2020. All logistic regression models adjusted for age (18–34, 35–54, 55–64, 65+), highest educational attainment (high school diploma or GED and below, some college, college and above), household income ($0–29,99, $30,000–59,999, $60000–99,999, $100,000 or more), gender (male, female), whether a household has children or not, employment status (employed, unemployed, others), and baseline pre-pandemic CES-D score. As previously mentioned, probable anxiety and depression was defined as scores on the overall scale ≥ 6, and probable depression (anxiety) was defined as scores on depression (anxiety) subscale ≥ 3. Demographic data were missing for some respondents in Wave 1 data, but we found no difference in PHQ-4 score between respondents with complete demographic data (n = 6,456) and respondents with incomplete demographic data (n = 476). As such, we treated the missing values as missing at random. Additionally, risk perception items were excluded from the final model as they did not affect the final results. Estimates from logistic regressions show that during the pandemic, Black Americans were significantly less likely than non-Black Americans to have probable anxiety and depression (AOR = 0.487; 95% CI = [0.308, 0.771]; P < 0.01); probable anxiety (AOR = 0.474; 95% CI = [0.319, 0.704]; P < 0.001); or probable depression (AOR = 0.522; 95% CI = [0.330, 0.824]; P < 0.01). More directly, the odds of Black Americans reporting symptoms of either anxiety or depression are about half that of non-Black Americans, and the differences are statistically significant. We also compared odds of having probable anxiety and/or depression among non-Black racial and ethnic groups during the pandemic (i.e., Whites, Hispanics, Asians, and non-Black others) but found no significant differences among them adjusting for the same covariates presented in Table 4 (results not shown). Odds ratios for covariates have expected signs: for instance, the odds of having probable anxiety and depression during the pandemic decline with age, household income, and increase with baseline CES-D scores and being unemployed or not working. With respect to the baseline CES-D score, our results indicate that for every one unit increase in the baseline CES-D score, the odds of having probable anxiety and depression, anxiety, or depression in a given month during the pandemic increases by about 35%, 31%, and 39%, respectively, consistent with findings from prior studies [31, 32].

**Table 3:** Prevalence of reporting symptoms for anxiety and depression, anxiety, and depression across subpopulation groups in Wave 1.

	Probable anxiety and depression	Probable anxiety	Probable depression
**Race, n (%)**			
Non-Blacks	738 (11.4%)	1,060 (16.72%)	679 (10.70%)
Blacks	52 (9.74)	72 (13.46%)	47 (8.79%)
**Age, n (%)**			
18–34	264 (18.57%)	351 (24.67%)	229 (16.08%)
35–54	320 (12.48%)	455 (17.74%)	294 (11.44%)
55–64	115 (8.23%)	184 (13.17%)	108 (7.73%)
65 plus	91 (6.10%)	143 (9.57%)	95 (6.37%)
**Education, n (%)**			
High school diploma or GED and below	205 (13.45%)	284 (18.61%)	200 (13.10%)
Some college	311 (12.19%)	401 (15.71%)	297 (11.62%)
College and above	274 (9.76%)	448 (15.95%)	229 (8.16%)
**Income, n (%)**			
$0–29,999	303 (18.25%)	373 (22.43%)	291 (17.50%)
$30–59,999	199 (11.31%)	268 (15.24%)	196 (11.13%)
$60–99,999	136 (8.11%)	233 (13.90%)	128 (7.63%)
$100,000 or more	150 (8.46%)	257 (14.50%)	109 (6.15%)
**Household, n (%)**			
Have no children	533 (11.37%)	750 (15.99%)	496 (10.57%)
Have children	257 (11.71%)	383 (17.45%)	230 (10.46%)
**Gender, n (%)**			
Female	556 (13.79%)	801 (19.86%)	475 (11.77%)
Male	234 (8.21%)	332 (11.64%)	251 (8.80%)
**Employment, n (%)**			
Employed	432 (10.46%)	665 (16.10%)	366 (8.85%)
Unemployed	109 (20.30%)	134 (24.95%)	104 (19.33%)
Others	249 (11.24%)	334 (15.06%)	256 (11.55%)

* p<0.10;

** p<0.05;

*** p<0.01

95% CIs are in parentheses.

**Table 4:** Adjusted odds ratios; cross-sectional analysis (Wave 1 data: March 10, 2020–March 30, 2020); outcome variables: Reporting symptoms for anxiety and depression (Column 1), anxiety (Column 2), and depression (Column 3).

	Anxiety and depression disorder	Anxiety disorder	Depression disorder
**Race (Reference is non-Black)**			
Black (March)	0.487***	0.474***	0.522***
	[0.308,0.771]	[0.319,0.704]	[0.330,0.824]
Black (April)	0.541***	0.563***	0.447***
	[0.379,0.772]	[0.410,0.773]	[0.304,0.658]
Black (May)	0.520***	0.571***	0.574***
	[0.351,0.771]	[0.406,0.801]	[0.404,0.815]
Black (June)	0.582**	0.559***	0.624**
	[0.376,0.900]	[0.373,0.836]	[0.411,0.946]
Black (July)	0.465***	0.439***	0.544***
	[0.291,0.744]	[0.295,0.653]	[0.355,0.833]
Black (August)	0.639**	0.675**	0.697*
	[0.421,0.970]	[0.459,0.991]	[0.464,1.046]
Black (September)	0.590**	0.557***	0.689*
	[0.386,0.900]	[0.366,0.848]	[0.466,1.020]
Black (October)	0.542***	0.535***	0.543***
	[0.342,0.859]	[0.354,0.811]	[0.347,0.848]
Black (November)	0.495**	0.495***	0.403***
	[0.282,0.869]	[0.297,0.825]	[0.221,0.737]
**Month (Reference is March)**			
April	0.950	0.872	1.310
	[0.611,1.477]	[0.601,1.264]	[0.831,2.063]
May	0.663	0.506***	0.991
	[0.403,1.089]	[0.333,0.770]	[0.593,1.657]
June	0.395***	0.381***	0.585*
	[0.227,0.689]	[0.235,0.618]	[0.330,1.035]
July	0.420***	0.350***	0.574*
	[0.236,0.746]	[0.210,0.585]	[0.328,1.006]
August	0.426***	0.311***	0.631
	[0.241,0.754]	[0.187,0.518]	[0.361,1.104]
September	0.545**	0.418***	0.733
	[0.328,0.907]	[0.257,0.682]	[0.436,1.232]
October	0.548**	0.397***	0.824
	[0.324,0.927]	[0.245,0.645]	[0.478,1.420]
November	0.452**	0.348***	0.647
	[0.241,0.849]	[0.200,0.606]	[0.338,1.238]
Covariates included	Yes	Yes	Yes
Covariates interacted with time variable	Yes	Yes	Yes
Observations	96,312	96,328	96,382
Pseudo R-squared	0.143	0.118	0.143

* p<0.10;

** p<0.05;

*** p<0.01.

95% CIs are in parentheses.

### Longitudinal analysis

Since the UAS COVID Survey collected data on the same individuals over time, we were able to assess whether differences in mental health outcome between Black Americans and non-Black Americans we observed in March persisted over time during the pandemic. In Table 5, we present our estimates of AORs from logistic regressions for having probable anxiety and depression in the first column; probable anxiety in the second column; and probable depression in the third column, for each month from March to November. Data are from all 17 waves of the UAS COVID Tracking Survey, fielded between Mar 10, 2020 and November 30, 2020. Clustered robust standard errors were used in logistic regression. Results are weighted. All logistic regression models adjusted for month of the year, age (18–34, 35–54, 55–64, 65+), highest educational attainment (high school diploma or GED and below, some college, college and above), household income ($0–29,99, $30,000–59,999, $60000–99,999, $100,000 or more), gender (male, female), whether a household has children or not, employment status (employed, unemployed, others), and baseline pre-pandemic CES-D score. Each of the demographic variables were interacted with the time variable to model potential change over time in the estimated coefficients. The coefficients of the demographic variables interacted with the time variable were omitted in Table 5. For comparison with logistic regressions, we also estimated AORs from a multilevel fixed-effects model using an unstructured covariance option, which allows for all variances and covariances to be distinctly estimated, but results are not shown; estimates from fixed-effects multilevel model estimation are quantitatively as well as qualitatively similar to those from logistic regression. The mean item non-response rate for the PHQ-4 across all waves (wave 1 through wave 17) is about 1% (S.D = 0.43%; Min = 0.44%; Max = 1.85%). We did not find any systematic relationship between the baseline CES-D score and item non-response for the PHQ-4. We conducted attrition analysis in the UAS panel and found that the average ann`ual attrition rate is about 9.4% and that that attrition was not selective by education, age, gender, income, race and ethnicity. The UAS panel is refreshed continuously throughout the year.

**Table 5:** Adjusted odds ratios; longitudinal analysis (Wave 1–17 data: March 10, 2020–November 30, 2020); outcome variables: Reporting symptoms for anxiety and depression (Column 1), anxiety (Column 2), and depression (Column 3).

	Probable anxiety and depression	Probable anxiety	Probable depression
**Race (Reference is non-Black)**			
Black (March)	0.487***	0.474***	0.522***
	[0.308,0.771]	[0.319,0.704]	[0.330,0.824]
Black (April)	0.541***	0.563***	0.447***
	[0.379,0.772]	[0.410,0.773]	[0.304,0.658]
Black (May)	0.520***	0.571***	0.574***
	[0.351,0.771]	[0.406,0.801]	[0.404,0.815]
Black (June)	0.582**	0.559***	0.624**
	[0.376,0.900]	[0.373,0.836]	[0.411,0.946]
Black (July)	0.465***	0.439***	0.544***
	[0.291,0.744]	[0.295,0.653]	[0.355,0.833]
Black (August)	0.639**	0.675**	0.697*
	[0.421,0.970]	[0.459,0.991]	[0.464,1.046]
Black (September)	0.590**	0.557***	0.689*
	[0.386,0.900]	[0.366,0.848]	[0.466,1.020]
Black (October)	0.542***	0.535***	0.543***
	[0.342,0.859]	[0.354,0.811]	[0.347,0.848]
Black (November)	0.495**	0.495***	0.403***
	[0.282,0.869]	[0.297,0.825]	[0.221,0.737]
**Month (Reference is March)**			
April	0.950	0.872	1.310
	[0.611,1.477]	[0.601,1.264]	[0.831,2.063]
May	0.663	0.506***	0.991
	[0.403,1.089]	[0.333,0.770]	[0.593,1.657]
June	0.395***	0.381***	0.585*
	[0.227,0.689]	[0.235,0.618]	[0.330,1.035]
July	0.420***	0.350***	0.574*
	[0.236,0.746]	[0.210,0.585]	[0.328,1.006]
August	0.426***	0.311***	0.631
	[0.241,0.754]	[0.187,0.518]	[0.361,1.104]
September	0.545**	0.418***	0.733
	[0.328,0.907]	[0.257,0.682]	[0.436,1.232]
October	0.548**	0.397***	0.824
	[0.324,0.927]	[0.245,0.645]	[0.478,1.420]
November	0.452**	0.348***	0.647
	[0.241,0.849]	[0.200,0.606]	[0.338,1.238]
Covariates included	Yes	Yes	Yes
Covariates interacted with time variable	Yes	Yes	Yes
Observations	96,312	96,328	96,382
Pseudo R-squared	0.143	0.118	0.143

* p<0.10;

** p<0.05;

*** p<0.01.

**Fig 1:**
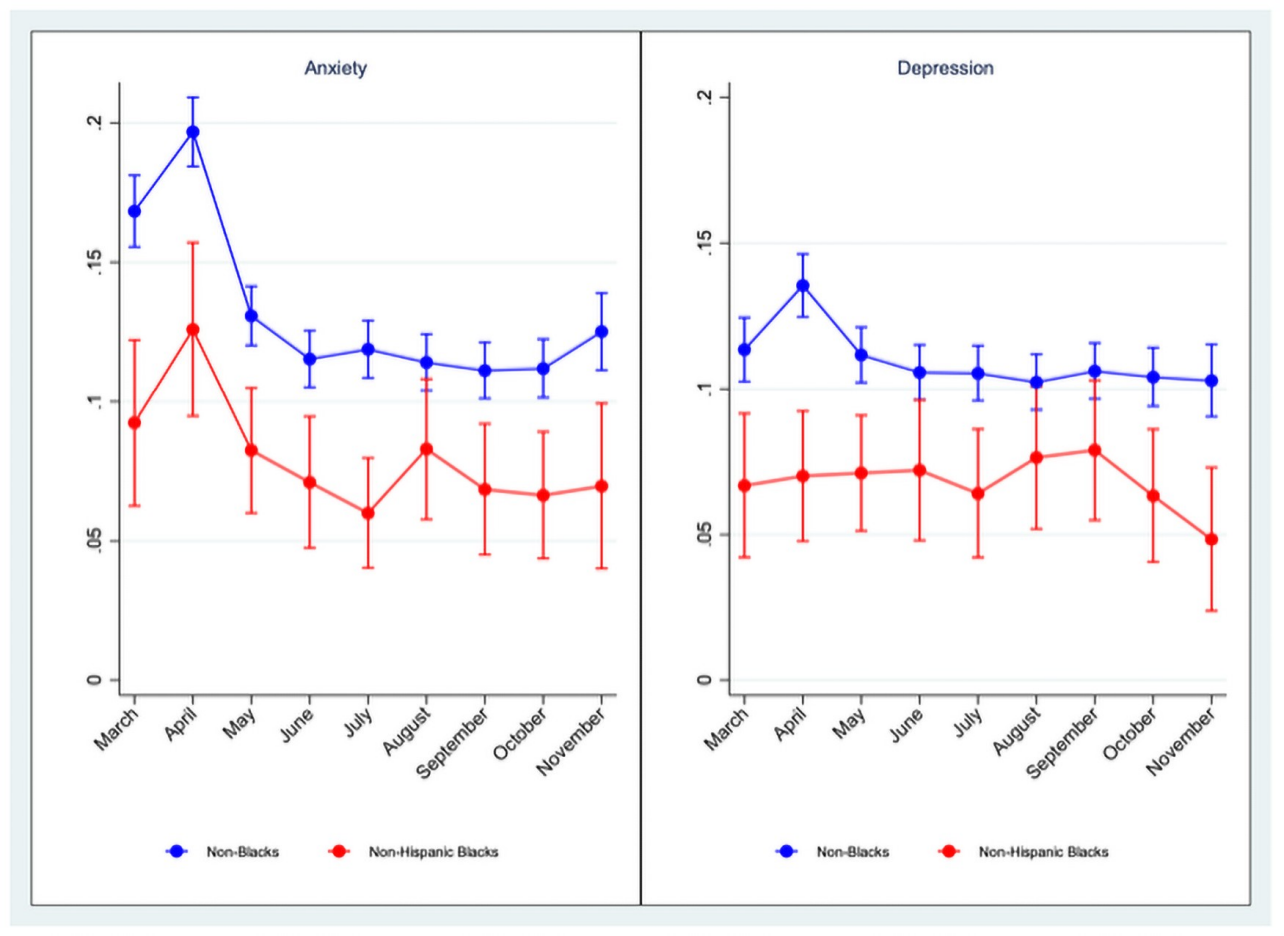
Predicted probabilities of reporting symptoms for anxiety and depression by month.

95% CIs are in parentheses. Fig 1 presents predicted probabilities of having probable anxiety and depression (together with 95% CIs) for each month from March 10 to November 30 for both Black and non-Black Americans separately. Fig 1 reveals that the monthly gap in mental health outcomes between Black and non-Black Americans remained throughout from March of 2020 to November of 2020, and that the gap in depression began to widen starting in September. The predicted probabilities adjusted for age (18–34, 35–54, 55–64, 65+), highest educational attainment (high school diploma or GED and below, some college, college and above), household income ($0–29,99, $30,000–59,999, $60000–99,999, $100,000 or more), gender (male, female), whether a household has children or not, employment status (employed, unemployed, others), and baseline pre-pandemic CES-D score. Stata’s “margins” command was used to generate predicted probabilities and “marginsplot” command was used to generate the above graphs after estimating the logistic regressions using the odds ratio (or) option.

Our results in Table 5 indicate that, as in March, Black Americans were significantly less likely than other racial groups to have probable anxiety and/or depression in the following months after March, even after controlling for age, education, household income, whether a household has children or not, gender, employment status, and (most importantly) baseline CES-D score. Again, the odds of Black Americans reporting symptoms in a given month are about half that of non-Black Americans, and the differences are statistically significant in all months.

Our results also indicate that overall probable anxiety and depression among the U.S. adult population was high in March and April, and then gradually began to improve starting in May. When broken down by type of disorder, the results also reveal that anxiety showed a higher degree of fluctuation from March to November in the U.S. adult population compared to depression. This may in part reflect differences in specificity for the measurements of depression (specificity of 0.92) and anxiety (specificity of 0.83; 19, 20).

## Discussion

Previous work has documented mental health disparities across racial and ethnic lines in times of disaster [33, 34]. Understanding how these differences manifest during the COVID-19 pandemic is critical to provide appropriate mental health interventions and implement effective policies. Thus, our goals in this study were to understand racial disparities in odds of having anxiety and depression among Black and non-Black Americans aged 18 and above when state lockdowns began in March of 2020, and to explore changes in this disparity over time between March and November.

We found the odds of Black Americans having depression and anxiety was lower on average throughout the pandemic than that of non-Black Americans. In contrast, we found no significant differences in mental health outcomes among non-Black racial and ethnic groups (e.g., Whites, Asians, Hispanics, and Others). We also found that the monthly gap in depression between Black and non-Black Americans began to widen in September of 2020. This is further supported by our finding that the baseline CES-D scores did not differ significantly between Black and non-Black Americans before the pandemic, but did differ significantly mid-pandemic by December of 2020. All analyses adjusted for sociodemographic variables and baseline pre-pandemic CES-D score. These findings are paradoxical given that Black Americans disproportionately face some types of mental health stressors compared to other racial and ethnic groups during the pandemic as previously discussed and that the physical health of Black Americans is significantly worse than non-Black Americans. To explore this result, we comment on our findings of mental health disparities during the COVID-19 pandemic in light of the mental health of Black Americans in general as well as mental health disparities during other times of disaster, drawing on findings from prior studies.

A few studies have considered racial and ethnic differences in mental health specifically during the pandemic, reporting heterogeneous findings. One study using a representative probability panel examined levels of psychological distress using the Kessler 6 scale before (in February 2019) and during the pandemic (in May 2020) among the same respondents. The authors found an increase in psychological distress among 12.8% of respondents, which was only more pronounced among Hispanic Americans compared to other racial and ethnic groups [31]. However, the research utilized a far smaller sample size than we analyzed (N = 1,870), so real differences between groups may not have been detectable. A second probability-based study measuring mental distress in the same way found no differences in prevalence by race/ethnicity between April and July of 2020, but again was limited by a small sample size (N = 1,337; 32). On the other hand, a longitudinal study in the UK found that racial and ethnic minorities experienced a greater increase in mental distress after the onset of the pandemic as measured by the General Health Questionnaire-12, possibly due in part to lockdowns and physical distancing [35]. A separate study in the Netherlands found no increase in anxiety or depression at all in the general population after the pandemic began and noted a stable employment situation relative to the U.S. which may play a large role [36]; the Netherlands’ universal healthcare system and better social safety net (relative to the US) may also explain this difference. With further investigation, these differences may shed some light on the factors contributing to the racial and ethnic disparities in mental health in the US. The similarity between mental health outcomes of Black Americans during COVID-19 and in other times of disaster varies. Black Americans remain more vulnerable to the physical effects of both the COVID-19 pandemic (as previously established) as well as disasters in general, but limited research on psychological impacts precludes any conclusive interpretations [33]. For example, hurricanes Ike and Katrina exacted a greater toll on Black persons, who disproportionately populated evacuation shelters as evacuees after Katrina and reported greater personal impact and loss of services after Ike compared to those who were White [34, 37]. As expected, Black Americans in hard-hit Mississippi counties reported higher levels of depression and psychological distress than White Americans in the aftermath of Hurricane Katrina [38], and Black Americans living in Texas after Hurricane Ike were more likely to meet criteria for PTSD compared to White and Hispanic Americans [34]. In these situations, racial disparities in psychological impact reflected those in physical impact, unlike what we observed during the COVID-19 pandemic. In contrast, a year after 9/11, Black persons living in New York City during the disaster were less likely than those who were White to meet criteria for major depression or have poor mental health [39, 40]; this is expected following our previous logic as the representation of Black lives lost during this disaster was less than that of the New York City population [41]. As discussed in the introduction, other research has shown that Black Americans experienced lower rates of anxiety and depression outside of the context of disasters before the pandemic, but had higher rates of serious conditions like PTSD or serious psychological distress [14, 15]. However, as previously discussed, we found no differences in depression (CES-D score) before the pandemic (although we had no ability to make comparisons with anxiety), and our main results showed lower rates for both anxiety and depression among Black Americans during the pandemic. Further research is required to determine whether racial and ethnic mental health disparities in terms of depression and anxiety in general differ from disparities in serious psychological distress during crises like COVID-19.

Marginal evidence indicates that Black Americans may systematically underestimate their risks compared to their counterparts. Previous research has found that Black Americans had the same odds as White Americans to believe that those of lower socioeconomic status and those who were Black were more likely to die of COVID-19 complications; however, Black Americans had lower odds than White Americans to hold this belief about those who were older and those who had chronic health conditions [42]. But this potential explanation was not supported by our data. We analyzed responses to risk perception questions in the UAS COVID-19 Tracking survey about the chance to get the coronavirus in the next three months, the chance of death if infected with the coronavirus, and the chance of running out of money because of the coronavirus in the next three months [28]. The data revealed that, instead of underestimating the risks, Black respondents perceived the same risk as non-Black respondents of getting infected with COVID-19 in the next 3 months and actually perceived a higher risk than non-Black respondents of death and running out of money due to COVID-19 in the next three months. This finding held even after controlling for demographics and baseline depression symptoms. Most importantly, our main findings were insensitive to whether we controlled for responses to the three risk perceptions, in addition to demographics and baseline depression symptoms (results not shown). Alternatively, some might argue that the mental health advantage of Black Americans over non-Black Americans is due to higher religious involvement among Black Americans and higher importance Black Americans place on religion, compared to non-Black Americans. However, the empirical evidence supporting the religious explanation has been limited [43].

Racial differences in reporting styles might also explain our results. The outcome measures used in this study are inherently subjective and thus are vulnerable to response scale biases. Different racial groups could use different response scales or attach different meanings to a verbal label when self-reporting their mental health [44–46]. For instance, Black Americans might have a higher standard over what it means to be mentally stressed, especially during crises like COVID-19 pandemic. If this is the case, then the differences in self-report of mental health reflects differences in response scale used by different racial groups rather than differences in underlying mental health conditions. Future research should investigate this possibility.

## Conclusion

Unlike any public health crisis in living memory, the COVID-19 pandemic has thrown the U.S. into upheaval. Black Americans have faced disproportionately high rates of illness, job losses, and death in its midst. The mental health of Black Americans thus remains a major concern as the pandemic and its effects unfold. Hence, in this study, we assessed the mental health of the U.S. adult population through probable anxiety and depression by race over time during the pandemic. We found no mental health differences among non-Black racial and ethnic groups, but discovered significantly better mental health among Black Americans in comparison. This result raises several questions given numerous prior reports suggesting that Black Americans are disproportionately affected by the pandemic in terms of mortality, hospitalization, and job loss. Understanding the significance of this result requires further research into how Black Americans think about their mental health and perceive health risks during public health crises.
